# Understanding the relationship between sleep and quality of life in type 2 diabetes: A systematic review of the literature

**DOI:** 10.1177/13591053221140805

**Published:** 2023-01-04

**Authors:** Bróna Laverty, Sreelakshmi Puthezhath Jayanandan, Sinéad Smyth

**Affiliations:** School of Psychology Dublin City University, Dublin, Ireland

**Keywords:** narrative synthesis, sleep disorders, sleep quality, type 2 diabetes, QoL

## Abstract

Living with type 2 diabetes (T2D) can elicit psychological distress and diminish quality of life (QoL) in patients. Research has also elucidated a link between sleep and quality of life. Thus, the current review aimed to clarify the relationship between sleep and QoL in T2D patients, and determine the prevalence of sleep problems in this cohort. A systematic search across four databases yielded 23 relevant studies, which were synthesized narratively. Between 17.8 and 79% of patients had sleep problems, and a direct, significant relationship was established between sleep and QoL. An indirect relationship between sleep and QoL was established through exacerbation of psychological factors and biological symptoms of T2D. Findings are clinically relevant and highlight the importance of screening for sleep problems during routine patient appointments. Future research should employ either longitudinal or prospective study designs to enable further understanding of the intricacies of this relationship.

## Introduction

Type 2 diabetes (T2D) is a chronic metabolic condition categorized by the body’s inability to adequately absorb insulin in the pancreas (Chatterjee et al., 2017), and an impaired ability to control blood glucose levels which can result in hyperglycaemia. Hyperglycaemia, considered the hallmark metabolic abnormality associated with T2D (Nathan et al., 2009), is characterized by elevated levels of glucose in the blood. Prolonged elevations in blood glucose can have detrimental health effects and may result in diabetes-related complications including retinopathy, neuropathy and diabetic nephropathy. Diabetes rates have increased such that it is now one of the most common chronic conditions in adults worldwide, at an estimated prevalence of 450 million, with T2D accounting for almost 90% of diabetes cases (Chatterjee et al., 2017). The remaining cases consist of those with type 1 diabetes (T1D), gestational diabetes or pancreatogenic diabetes.

Unlike the autoimmune condition T1D, the development of T2D is attributed to several lifestyle related risk factors. These include obesity and physical inactivity (American Diabetes Association, 2017), as well as increased age, genetics and family history (Boles et al., 2017). There also appears to be a link between sleep and T2D, with obstructive sleep apnoea (OSA), a sleep disorder pervasive among overweight and obese adults (Wu et al., 2014), identified as a modifiable risk factor affecting insulin resistance which may influence the development of T2D (Pamidi and Tasali, 2012). Furthermore, quantity and quality of sleep have been found to predict the development of T2D (Cappuccio et al., 2010).

A diagnosis of T2D brings with it physical and psychological challenges which can negatively impact quality of life (QoL). Many patients living with the condition feel diabetes powerfully affects their lives, and most feel burdened by the manifold demands of their disease (Rubin, 2000). A new diagnosis often involves altering behaviours in line with diabetes self-management, which is a crucial component in adapting to life with the condition. Self-managing T2D includes adopting lower fat diets, and regular exercising (Gomersall et al., 2011), as well as checking blood sugar levels. Due to the, sometimes severe, lifestyle changes implemented to prevent diabetes-related complications, this patient group may be particularly vulnerable to psychological distress. Given the substantial disease burden T2D imposes on both an individual and society, research has focused on the factors that have prevented patients living relatively well despite their condition by negatively impacting their QoL, one of which is sleep.

### QoL and T2D

QoL is defined as overall general well-being that comprises objective descriptors and subjective evaluations of physical, emotional, material and social well-being (Felce and Perry, 1995). Health-related QoL (HRQoL) in T2D patients is an efficacious measure evaluating the impact of the disease and its treatment on individuals and health care costs (Luyster and Dunbar-Jacob, 2011). The term HRQoL comprises aspects of QoL shown to affect physical or mental health (Trikkalinou et al., 2017). Improving HRQoL is at the centre of focus in the treatment/management of all chronic health conditions (Onu et al., 2022). Lower reported HRQoL was associated with higher rates of mortality among T2D patients (Kleefstra et al., 2008). Individuals also reported experiencing emotional burdens and poor sleep quality in response to prolonged requirements set out by the long-term self-management involved with living with T2D (Dong et al., 2020). Further research also demonstrated a relationship between health anxiety and trait anxiety, fear of diabetes complications and lower physical quality of life in diabetes patients (Janzen Claude et al., 2013). When considering the impact the diagnosis of T2D can have on patient QoL, this elevates the importance of investigating factors that may perpetuate negative effects of the condition.

### Sleep and T2D

Sleep is a natural physiological process which is fundamental for psychological and biological functioning, including glucose metabolism in T2D (Lee et al., 2017). Sleep plays an important role in well-being including in individuals with chronic illness (Zhu et al., 2018). Sleep problems often occur concurrently with T2D, with prevalence estimated between 42 and 76.8% (Gupta and Wang, 2016; Nefs et al., 2015; Zhu et al., 2018). T2D patients are at increased risk of sleep problems and sleep disorders (Chasens et al., 2013). Sleep problems encapsulate sleep disturbances and all disorders which cause difficulty initiating and maintaining sleep (Cho, 2020), and may extend to issues with sleep duration and quality. Sleep disorders are diagnosed using strict criteria, and are classified using distinct categories, such as insomnia and sleep apnoea (Zhu et al., 2018), while sleep disturbances do not require a diagnosis and can be experienced by anyone at any point in their life. Sleep disturbances are associated with increased risk for poor physical and mental health (Colten and Altevogt, 2006), which are crucial to QoL.

Previous research has alluded to the complex, bidirectional nature of the relationship between sleep and T2D. Sleep problems may be both a precursor to, and a consequence of, the development of T2D (Cappuccio et al., 2010; Chasens et al., 2013; Pamidi and Tasali, 2012). Studies of healthy young adults undergoing recurrent partial sleep restriction reported marked alterations in glucose metabolism including decreased glucose tolerance and insulin sensitivity (Spiegel et al., 2005). These findings illustrate the critical role of sleep in the maintenance of normal endocrine function. Independent of confounding factors, both short and long sleep durations may increase the risk of developing diabetes, (Yaggi et al., 2006). Sleep deficits can also foster metabolic syndrome that culminates in sleep disorders like restless leg syndrome and sleep apnoea, which in turn lead to poor sleep quality (Martins et al., 2008). Reviews and meta-analyses in the area of sleep and T2D have focused on the impact of fatigue on self-management and quality of life (Kuo et al., 2022), and the impact of sleep quality and duration on glycaemic control (Lee et al., 2017). However, the magnitude and diversity of sleep problems experienced by T2D patients and their diminished QoL demands an overall assessment of the sleep-QoL relationship in this population.

### Review purpose

Despite a high prevalence of sleep problems reported among T2D patients, to our knowledge, no previous systematic review has examined the relationship between sleep in a broad context and QoL in patients with T2D. Clarity on the nature of this relationship is essential for the implementation of targeted interventions that could improve sleep quality and improve patient QoL. The objective of our review is to bridge this gap in the literature and clarify the relationship between sleep and QoL in patients with T2D. A secondary objective is to illustrate the prevalence rate of sleep problems and symptoms of disordered sleep among T2D population within included studies, which would quantify the number of patients who may be vulnerable to poorer QoL.

## Method

### Search strategy

A systematic search of the literature was carried out on 3rd January 2022 across four electronic databases; Medline via EBSCO, PsycINFO, Cumulative Index to Nursing and Allied Health (CINAHL) and Web of Science. These databases were chosen because they were health discipline-specific, while Web of Science offered an interdisciplinary database. Our search strategy was devised through trial and error and scoping results on the selected databases. Following consultation with a librarian, a final set of search terms and Boolean operators were determined. These included ‘sleep’ AND ‘quality of life’ AND ‘diabetes’. ‘Sleep’ was chosen as a broad text term so as not to restrict search results as we were interested in all aspects of sleep. Free text terms relating to ‘quality of life’ (e.g., ‘health-related quality of life’ AND ‘diabetes-related quality of life’) were generated and included in the search. The decision was made to use ‘diabetes’ instead of ‘type 2 diabetes’ because our review considered studies with both T1D and T2D if results and findings were distinguished between both cohorts. The search terms are in the Supplemental Files.

### Eligibility criteria

The inclusion criteria for our review were constructed in accordance with the PICOS framework for framing and reporting review criteria. Empirical studies were included if; 1) participants were over eighteen, 2) participants had T2D, 3) they quantitatively measured the relationship between sleep and QoL, 4) used reliable and validated measures of QoL, 5) published in English, 6) were peer reviewed. Studies were examined for evidence of a direct or indirect relationship between sleep and quality of life. Specifically, if the research design explored a direct association between the two variables or else observed interaction effects of sleep on quality of life through a third predictor or independent variable. The indirect effect of sleep on quality of life through the exacerbation of symptoms of ill-health was also considered. Studies were excluded if they 1) contained a sleep intervention, 2) QoL was not the main outcome measure, 3) the analysis did not examine the relationship between sleep and QoL, 4) studies wherein the sample composed of patients with diabetes that was not T2D, 5) they contained qualitative research only. A detailed summary of the inclusion and exclusion criteria can be viewed in the Supplemental Files.

### Risk of bias

To minimize potential bias, the review team independently assessed each study’s suitability for inclusion. Results from each database were transferred to ‘Zotero’ reference manager software (Corporation for Digital Scholarship, 2021), and then uploaded to ‘Covidence’ (Veritas Health Innovation, 2021) where duplicates were removed. Covidence, as a web-based software developed specifically for the process of systematic reviews, enabled dual screening by the review team throughout the title and abstract and full-text screening process. Data extraction and quality appraisal processes were also independently carried out by the review team. The review team met at each stage to discuss their decision in relation to studies to be included to ensure they were in agreement. When conflict arose, disagreements were resolved by meeting and going through decisions to achieve consensus.

We followed the PRISMA framework, a standardized guide for reporting systematic reviews and meta-analyses. Through use of this framework, the risk of bias was kept to a minimum. Furthermore, to minimize the risk of bias from the outset, we developed and followed a review protocol outlining all aims, objectives and inclusion and exclusion criteria. .

### Data extraction

Data were extracted in accordance with PRISMA guidelines, and recorded in a Microsoft Excel data extraction table. Extracted data included bibliographic codes like author, year of study and country of origin. Substantive codes recorded included study design, sample size, sleep measure, QoL measure, key findings, statistical significance, method of analysis, quality assessment rating, length of time since T2D diagnosis and mean age.

### Quality assessment

We used a critical appraisal tool adapted from (Dunne et al., 2017) to evaluate the quality of the quantitative studies. This consists of 12 items designed to evaluate the risk of bias inherent in research studies. The items can be assigned an answer of ‘yes’, ‘partial’, ‘no’, ‘don’t know’, which translated to scores 2, 1, 0, and 0, respectively. Studies were assessed out of a possible score of 24, with the higher the score indicating a higher quality study. The critical appraisal of included studies was carried out separately by both reviewers who then met to go through studies in tandem, ensuring the review team were in agreement with the quality ratings assigned. During this meeting, consensus was achieved.

### Synthesis of results

A detailed map of the literature was executed and reviewed concurrently with the data extraction table to determine the most appropriate method of data synthesis. Due to the heterogeneity apparent across QoL measures, a narrative synthesis was conducted which involved carefully examining the data extraction table for patterns and relationships.

## Results

**Figure 1.**
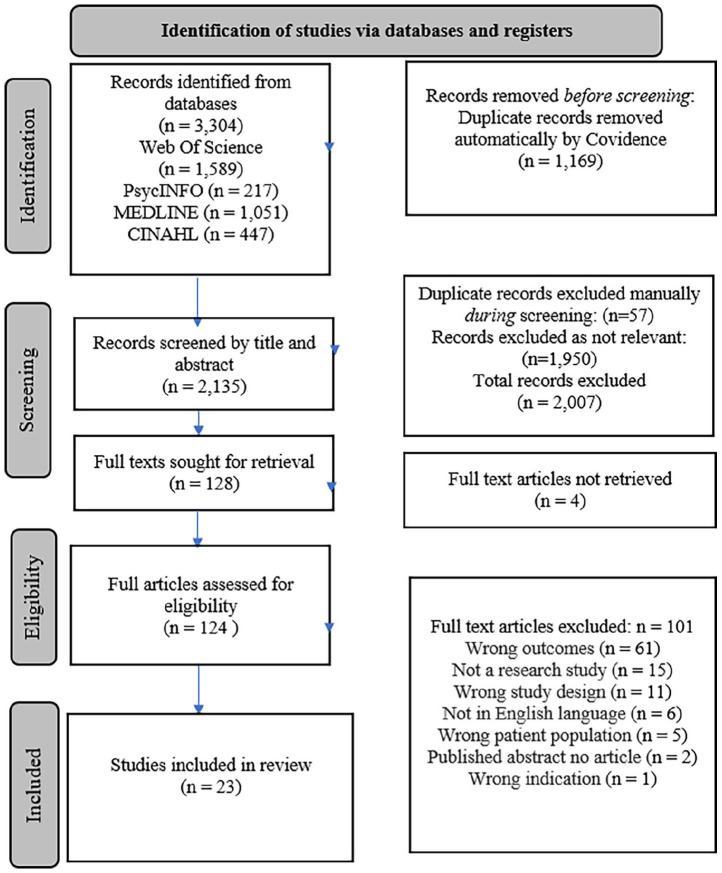
PRISMA flowchart.

The search yielded 3304 studies. Following the removal of duplicates in Covidence, 2076 studies remained and were independently assessed for suitability by the review team. A total of 128 were deemed as potentially relevant and moved to the full-text stage. These studies were reviewed against predefined inclusion and exclusion criteria to determine suitability, after which 23 studies remained for inclusion in our review (see Figure 1)

### Study characteristics

The publication dates of included studies ranged from 2007 to 2021. Participants were 8570 adults (female = 4489; male = 4081) aged between 18 and 97, with T2D. Sample sizes ranged from 50 to 944. Five studies did not include information regarding time since T2D diagnosis (Chasens et al., 2014; Narisawa et al., 2017; Seligowski et al., 2013; Yücel et al., 2015; Zeng et al., 2018). Of the remaining studies, length of time since T2D diagnosis ranged from 5.6 to 13.5 years. Study designs employed included cross sectional (*n* = 15), secondary data analysis (*n* = 6), descriptive (*n* = 1) and observational (*n* = 1). Study locations were China (*n* = 6), America (*n* = 3), Brazil (*n* = 2), Japan (*n* = 2), India (*n* = 2), Canada (*n* = 1), Italy (*n* = 1), Iran (*n* = 1), France (*n* = 1), UAE (*n* = 1), Croatia (*n* = 1), Turkey (*n* = 1) and Spain (*n* = 1). The characteristics of the included studies are displayed in Table 1.

**Table 1.** Characteristics of included studies.

Author and year	Study design	Study location	*N*	Mean length of diagnosis	Sleep measure	QoL measure	Findings	Quality
Azharuddin et al. (2020)	Cross sectional	India	300	7.40 ± 6.75	PSQI	EQ 5D	Poor sleepers had lower HRQoL. Patient FPG and PP were higher among poor sleep quality groups compared to good sleep quality groups.	20
Bani-issa et al. (2018)	Cross sectional	UAE	268	N/A	PSQI	WHOQOL-BREF	Global PSQI score was a strong independent predictor of global HRQoL. Hba1c was not significantly related to HRQoL.	21
Bironneau et al. (2017)	Cross sectional	France	126	9.0 ± 7.7	Polysom-nography	SF-36 (French Version)	OSA severity was associated with lower scores across three domains of HRQoL and higher systolic blood pressure.	19
Chasens et al. (2014)	Secondary analysis	USA	116	N/A	PSQI	SF-36v2	Impaired sleep quality was significantly associated with lower scores on the physical component summary (PCS) and mental component summary (MCS) (*p* = 0.001)	20
Daniele et al. (2013)	Observational	Brazil	200	11.7 ± 7.5	PSQI	SF-36	There was a significant difference between RLS group and control in five SF-36 domains and hypertension.	19
Dong et al. (2020)	Cross sectional	China	944	5.6 ± 5.1	PSQI	DSQL	There was a significant interaction between poor sleep quality and anxiety symptoms; this combined effect significantly reduced QoL	22
Gabric et al. (2018)	Cross sectional	Croatia	466	13.2 ± 9.4	ESS, STOP	SF-36	High-risk OSA group had significantly lower SF-36 scores. The presence of arterial hypertension and asthma was higher in high risk group.	17
Hashimoto et al. (2020)	Cross sectional	Japan	342	13.54 ± 10.59	DSM Sleep Disorders	SF- 36v2, EQ-5D	The PCS, MCS, EQ-5D of T2D patients with sleep disorders were lower than those without	16
Jain et al. (2017)	Cross sectional	India	50	5.6 ± 2.35	ISI	WHOQOL- BREF	Patients with insomnia scored lower than those without insomnia in all four domains of QOL. These findings were statistically significant for all domains (*p* < 0.01)	18
Johnson et al. (2017)	Secondary analysis	Canada	168	13 ± 9	Actigraphy	EQ-5D-5L, SF- 12.	Lower sleep efficiency was significantly associated with lower scores on both PCS and MCS scores of the SF-12 and longer total sleep time was associated with lower PCS scores	20
Li et al. (2019)	Secondary analysis	China	302	5.6 ± 5.1	PSQI	DQoL	Trait anxiety at baseline had a significantly negative prediction of patients’ QOL. Impaired sleep quality negatively influenced patients’ QOL only at baseline	20
Lou et al. (2015)	Cross sectional	China	944	5.6 ± 5.1	PSQI	DSQL	Poor sleepers had significantly poorer DSQL. Depressive and anxiety symptoms had a positive relationship with DSQL.	11
Luyster and Dunbar-Jacob (2011)	Secondary analysis	USA	300	9.4 ± 7.3	PSQI	SF-36, DQoL	Poor sleepers had poorer QoL scores and more depressive symptoms and comorbidities than good sleepers.	21
Merlino et al. (2010)	Secondary analysis	Italy	124	12.3 ± 9.86	IRLS Rating Scale	SF-36 (Italian Version)	RLS+ patients had significantly lower scores across SF-36 domains. The IRLS score showed a significant inverse correlation with vitality (*p* = 0.02), mental health (*p* = 0.04), and MCS (*p* < 0.001)	20
Modarresnia et al. (2018)	Cross sectional	Iran	210	7.8 ± 4.89	PSQI	EQ-5D	Those with RLS had a significantly lower QoL score compared to patients without RLS (*p* = 0.009)	19
Naranjo et al. (2020)	Cross sectional	Spain	130	11.9 ± 3.14	(MOS) Sleep Scale	SF-12-v2	A decrease in mental and physical QoL was associated with sleep disorders.	20
Narisawa et al. (2017)	Cross sectional	Japan	622	N/A	PSQI	SF-8	Poor sleepers with T2D had lower mental and physical component summary scores (MCS and PCS) than general population.	21
Seligowski et al. (2013)	Cross sectional	USA	86	N/A	PSQI	DQoL	PSQI was significantly related to DQOL. The PSQI had an indirect effect on relationship of anxiety and DQOL and depression and DQOL	22
Vieira et al. (2008)	Cross sectional	Brazil	105	9.9 ± 7.7	PSQI	WHOQOL-BREF	Poor sleepers had significantly lower QoL scores than good sleepers. A negative correlation was found between HbA1c and FPG and sleep duration and efficiency.	16
Yücel et al. (2015)	Descriptive	Turkey	81	N/A	PSQI	SF-36 (Turkish Version)	A moderate negative correlation was found between the Global PSQI score and sub-dimensions of SF 36. A highly significant positive correlation was found between PSQI global score and BDI and BAI (*p* < 0.05)	11
Zeng et al. (2018)	Cross sectional	China	798	N/A	sleep duration and rated sleep quality	DSQL (Chinese version)	The odds ratios of better QoL increased as sleep quality improved.	15
Zhang et al. (2016)	Secondary analysis	China	944	5.6 ± 5.1	PSQI	DSQL	Longer sleep duration and good sleep quality were significantly associated with an improvement in QOL. The combined effect of poor sleep quality and depressive symptoms produced reductions in DSQL	21
Zhao et al. (2016)	Cross sectional	China	944	5.6 ± 5.1	PSQI	DSQL	Each domain and total DSQL scores of poor sleep quality group were higher than the good sleep quality group. Each domain and total DSQL scores of poor sleep quality and depression group were higher than the normal group (*p* < 0.01)	14

### Sleep measures

Seven measures that assessed sleep disturbances, sleep disorders and sleep quality in T2D patients were used in the included studies (Table 1) – The Pittsburgh Sleep Quality Index (PSQI; *n* = 15), Epworth Sleepiness Scale (ESS; *n* = 1), Insomnia Severity Index (ISI, *n* = 1), Actigraphy (*n* = 1), Polysomnography (*n* = 1), International RLS Rating Scale (*n* = 1) and the STOP questionnaire (*n* = 1). One study enlisted questions regarding usual sleep duration per day and asked participants to rate their sleep quality; their responses were then used to assess sleep components (Zeng et al., 2018).

### Quality of life (QoL) measures

There was considerable diversity in the scales used to assess QoL in T2D patients. The Medical Outcomes Study 36-Item Short Form (SF-36; *n* = 8) and the Diabetes Specific Quality of Life scale (DSQL; *n* = 5) were the most used scales. The remaining scales were; European Quality of Life-5 Dimensions questionnaire (EQ-5D; *n* = 4), World Health Organization Quality of Life- 26-Item (WHOQOL-BREF; *n* = 3), Diabetes Quality of Life (DQoL; *n* = 3), Medical Outcomes Study 12-Item Short-Form Health Survey version 2 (SF-12; *n* = 2), Medical Outcomes Study 8-item Short Form Health Survey (SF-8, *n* = 1). Three studies used more than one scale to assess QoL among T2D patients (Hashimoto et al., 2020; Johnson et al., 2017; Luyster and Dunbar-Jacob, 2011).

### Study quality

According to the quality appraisal tool (Dunne et al., 2017), the majority of included studies (18) were good quality, while five were acceptable quality (see Supplemental Files). Within poorer quality studies, there was a lack of clarity regarding sample characteristics, and insufficient detail provided regarding study limitations. Other common characteristics across acceptable quality studies were the absence of a control group and a lack of detail regarding details of non-respondents. Several studies also lacked justification for the recruited sample size.

### Sleep and QoL

With the exception of Naranjo et al. (2020) and Seligowski et al. (2013), all studies reported the prevalence of sleep problems among their recruited sample. The prevalence of sleep problems reported ranged from 17.8 to 79%. Sleep problems encapsulated participants categorized as ‘poor sleepers’ and those that had sleep symptoms characteristic of sleep disorders and disturbances.

### Sleep disorders and QoL

Eight studies investigated the impact of sleep disorders on QoL (Bironneau et al., 2017; Daniele et al., 2013; Gabric et al., 2018; Hashimoto et al., 2020; Jain et al., 2017; Merlino et al., 2010; Modarresnia et al., 2018; Naranjo et al., 2020). Hashimoto et al. (2020) reported that patients with sleep disorders scored significantly lower across several dimensions of QoL (*p* < 0.001). This was supported by Naranjo et al. (2020), who found that a decrease in mental QoL was associated with sleep disorders (*p* < 0.01).

#### Restless leg syndrome

Three studies investigated RLS (Daniele et al., 2013; Merlino et al., 2010; Modarresnia et al., 2018). Results demonstrated relative consensus regarding the impact of RLS on QoL. Daniele et al. (2013) reported a significant difference between patients with RLS and without RLS across domains of the SF-36 (*p* < 0.01). Similarly, Merlino et al. (2010) found that patients with RLS had significantly lower scores across SF-36 domains, with IRLS score showing a significant inverse correlation with the mental component summary (MCS) domain (*p* < 0.001). Modarresnia et al. (2018) also reported that patients with RLS had a significantly lower QoL score compared to patients without RLS (*p* = 0.009).

#### Obstructive sleep apnoea

Two studies assessed the impact of OSA on QoL in T2D patients (Bironneau et al., 2017; Gabric et al., 2018). Bironneau et al. (2017) found that OSA severity was associated with lower scores across three domains of HRQoL, including vitality (*p* = 0.02), role functioning (*p* = 0.01) and social functioning (*p* = 0.04). Gabric et al. (2018) also found that patients with a high-risk of OSA exhibited significantly lower scores across dimensions of SF-36 compared to those with a low-risk (*p* = 0.001).

#### Insomnia

Only one study assessed the impact of insomnia on QoL in T2D patients (Jain et al., 2017). Jain et al. (2017) reported that patients with insomnia scored lower than those without insomnia on all four domains of QoL, including physical and psychological health and social relationships. These findings were statistically significant for all domains (*p* < 0.01).

### Non-clinical sleep disturbances and QoL

Two studies assessed the impact of non-clinical sleep disturbances on QoL (Johnson et al., 2017; Zeng et al., 2018). Johnson et al. (2017) reported that lower sleep efficiency was significantly associated with lower scores on both the PCS and MCS domains of the SF-36, while longer sleep time was associated with lower PCS scores. Similarly, Zeng et al. (2018) found that the odds ratios of better QoL increased as sleep quality improved in all the models in which sleep quality was a predictor.

### Sleep quality and QoL

Fifteen studies examined the impact of sleep quality on QoL in T2D patients (Azharuddin et al., 2020; Bani-Issa et al., 2018; Chasens et al., 2014; Dong et al., 2020; Li et al., 2019; Lou et al., 2015; Luyster and Dunbar-Jacob, 2011; Narisawa et al., 2017; Seligowski et al., 2013; Vieira et al., 2008; Yücel et al., 2015; Zhang et al., 2016; Zhao et al., 2016). These studies also investigated confounding factors that may contribute to sleep symptoms and negatively impact QoL, including psychological factors, biochemical factors, and comorbidities.

### Factors associated with sleep quality and QoL

#### Psychological factors

Psychological factors associated with sleep and QoL were investigated across nine studies (Dong et al., 2020; Li et al., 2019; Lou et al., 2015; Luyster and Dunbar-Jacob, 2011; Narisawa et al., 2017; Seligowski et al., 2013; Yücel et al., 2015; Zhang et al., 2016; Zhao et al., 2016). Dong et al. (2020) found a significant interaction between poor sleep quality and anxiety symptoms, and this combined effect significantly reduced QoL scores 6.09-fold. Furthermore, participants with poor sleep quality and anxiety symptoms had a significantly increased risk of reduced health status as measured by DSQL scores, compared with those with neither poor sleep quality nor anxiety (*p* < 0.001). Seligowski et al. (2013) reported similar findings, with PSQI scores demonstrating an indirect effect on the relationship between anxiety and diabetes-specific QoL and depression and QoL (*p* < 0.001). They also demonstrated that the PSQI was significantly related to DQOL (B = −1.15, *p* = 0.003). When comparing T2D patients to the general population, Narisawa et al. (2017) found poor sleepers in both groups had lower mental and physical component summary scores (MCS and PCS) from the SF-8. However, poor sleepers in the T2D group had lower PCS than poor sleepers in the general population group. Two studies also demonstrated a significant relationship between poorer sleep quality and lower DSQL scores (Lou et al., 2015; Zhao et al., 2016). Zhao et al. (2016) found a significant difference in DSQL scores between participants experiencing poor sleep quality compared to a control (*p* < 0.01).

Lou et al. (2015) further evidenced the influence of psychological factors on sleep and quality of life in their finding that participants with depressive and anxiety symptoms had a positive relationship with DSQL. Yücel et al. (2015) also reported a significant negative association between QoL and severity of depression and anxiety (*p* < 0.05). They found a moderate negative association between PSQI global score and sub-dimensions of SF-36. However, a highly significant positive correlation was found between PSQI global score and BDI and BAI scores (*p* < 0.05). Zhang et al. (2016) reported a statistically significant difference in the prevalence of depressive symptoms between patients with good QoL and poor QoL (*p* < 0.01). This was also the case with rates of poor sleep quality between groups of patients with poor and good QoL (*p* < 0.01). The combined effect of poor sleep quality and depressive symptoms produced reductions in DSQL, although this effect was felt to a greater degree by women than men (Zhang et al., 2016). Li et al. (2019) concluded that trait anxiety at baseline had a significantly negative prediction of patient QoL at baseline, 6 months, and 12 months (all *p* < 0.001), while impaired sleep quality negatively influenced QoL only at baseline (*p* = 0.039). Finally, Luyster and Dunbar-Jacob (2011) reported that patients with a previous history of anxiety scored lower in the MCS domain of the SF-12, compared to those without. In line with previous research, their study also demonstrated a significant association between sleep quality and QoL, with poor sleep quality associated with lower DQoL total scores and lower scores on the satisfaction, impact and diabetes-related worry subscales of the DQoL scale (all *p* < 0.001).

#### Biochemical factors

Three studies (Azharuddin et al., 2020; Bani-Issa et al., 2018; Vieira et al., 2008), examined the impact of biochemical factors such as Haemoglobin ba1c (Hba1c), fasting plasma glucose (FPG) and postprandial glucose (PP) on sleep and QoL. Azharuddin et al. (2020) concluded that T2D patients categorized as ‘poor sleepers’ had significantly lower HRQoL (*p* < 0.001). They reported that EQ-5D index and EQ-5D VAS were significant independent predictors of sleep quality (*p* < 0.05, *p* < 0.01), respectively. They also found that mean FPG and PP were significantly higher among poor sleep quality groups compared to good sleep quality groups (*p* < 0.05). Conversely, difference in Hba1c was not significant between good sleepers and poor sleepers (*p* = 0.0168), (Azharuddin et al., 2020). Similar findings were demonstrated by Bani-issa et al. (2018), who reported that global PSQI was a strong independent predictor of global HRQoL (*p* < 0.001). Hba1c was not controlled for during the regression analysis, as it was not significantly related to HRQoL (*p* = 0.200). Vieira et al. (2008) observed a significant difference between patients with good sleep quality and poor sleep quality in the general QoL and psychological domains of the WHOQOL-BREF (*p* < 0.005 and *p* < 0.01, respectively). With respect to biochemical factors, they reported a negative correlation was observed between HbA1c and sleep duration and efficiency (*p* < 0.01 and *p* < 0.005, respectively), while a negative correlation was observed between FPG and sleep duration (*p* < 0.05) and sleep efficiency (*p* < 0.02).

#### Comorbid conditions

The relationship between comorbid conditions and sleep was examined in five studies (Bironneau et al., 2017; Daniele et al., 2013; Gabric et al., 2018; Luyster and Dunbar-Jacob, 2011; Naranjo et al., 2020). Naranjo et al. (2020) found that in patients with diabetic neuropathy, more sleep disorders (*p* = 0.02) were negatively associated with the PCS component of the SF-12. Furthermore, optimal sleep (7–8 hours) was more frequent in pain-less patients. Luyster and Dunbar-Jacob (2011) found a statistically significant difference in the number of comorbidities and depressive symptoms for good sleepers compared to poor sleepers (both *p* ⩽ 0.001). Poorer sleep quality was associated with a higher number of comorbidities, greater number of diabetic complications and higher levels of depressive symptoms. Gabric et al. (2018) reported that the prevalence of arterial hypertension and asthma was significantly higher in subjects with high risk for OSA. Furthermore, the group with high risk for OSA exhibited higher prevalence of depression and GERD. When considering the impact of sleep disorders, Bironneau et al. (2017) found that OSA severity was associated with significantly higher systolic blood pressure (*p* = 0.03). Daniele et al. (2013) found a statistically significant difference of hypertension between patients with RLS and without RLS with hypertension (*p* = 0.02).

## Discussion

The demands of living with T2D and engaging in self-management can elicit psychological distress. Not only can this diagnosis inhibit psychological functioning, but effects can also extend to biological functions such as sleep. Extant research has elucidated the bidirectional association between sleep and T2D. Thus, our aim was twofold; to aggregate empirical research to clarify the nature of the relationship between sleep and QoL, and to highlight the prevalence of sleep problems in this patient cohort. Our findings revealed a significant relationship between sleep disturbances, sleep disorders and sleep quality and QoL among T2D patients. Our synthesis presents several key findings that may be used to inform future research and treatment.

A high prevalence of sleep problems was apparent across included studies, with between 17.8 and 79% of patients surveyed categorized as ‘poor sleepers’ or exhibiting sleep symptoms characteristic of sleep disorders. This is consistent with previous research estimating prevalence of sleep problems in T2D patients between 46 and 76% (Gupta and Wang, 2016; Nefs et al., 2015; Zhu et al., 2018). We also found evidence of a direct relationship between sleep and QoL in this patient cohort. The presence and severity of sleep disorders like RLS, OSA and insomnia was associated with lower scores across several HRQoL domains in T2D patients (Bironneau et al., 2017; Daniele et al., 2013; Jain et al., 2017; Merlino et al., 2010; Modarresnia et al., 2018). Gabric et al. (2018) also found evidence of a predictive relationship between sleep and QoL. Sleep quality was also a strong independent predictor of global HRQoL (Bani-issa et al., 2018). Extant literature illustrates the influential role disordered sleep has on health, with a recent systematic review highlighting sleep disorders can have a detrimental effect on the health, mood and QoL of T2D patients (Khandelwal et al., 2017). In this review, a significant relationship was also determined between sleep quality and QoL, with patients categorized as ‘poor sleepers’ more likely than their counterparts to score poorly across several QoL domains (Lou et al., 2015; Vieira et al., 2008; Zhao et al., 2016). This is comparable to previous research demonstrating a similar link between fatigue and self-management (Kuo et al., 2022), and sleep quality and duration on glycaemic control (Lee et al., 2017), which are both critical to living well with diabetes.

An indirect relationship between sleep and QoL was also evident within the majority of included studies. Depression and anxiety are common among T2D patients, with Thomas et al. (2003) highlighting their prevalence at 36% among patients. More recent research has demonstrated a marked increase in the frequency, with between 48.27 and 57.9% suffering from anxiety and between 43.5 and 56.1% suffering with depression (Khuwaja et al., 2010; Sun et al., 2016; Tovilla-Zarate et al., 2012). Our findings highlight a combination of poor sleep quality and psychological factors such as depression and anxiety-worsened patient reported QoL (Dong et al., 2020; Seligowski et al., 2013; Zhang et al., 2016). Furthermore, sleep quality showed a partial indirect effect on the relationship between depression and anxiety symptoms and diabetes QoL (Dong et al., 2020; Seligowski et al., 2013). These findings suggest that while sleep quality is solely influential on QoL, it can also exacerbate depressive and anxiety symptoms that may culminate to reduce patient QoL. Existing behavioural interventions exist for the treatment of depression disorders and poor sleep quality, which may prove efficacious in alleviating symptoms in patients (Manber et al., 2008; Zhang et al., 2016).

To reduce the risk of the development of diabetes-related complications, it is important that patients maintain good glycaemic control (Stolar, 2010). Better glycaemic control is perceived to be an important method to reduce the risk of complications and improve HRQL (Bani-issa et al., 2018; Lou et al., 2015). We found that patient Hba1c can be significantly impacted by sleep duration and efficiency, with evidence demonstrating a negative association (Vieira et al., 2008). Furthermore, fasting plasma glucose and postprandial glucose were found to be negatively associated with sleep quality (Azharuddin et al., 2020) and sleep efficiency (Vieira et al., 2008). Given the relationship between diabetes complications and glycaemic control, there is a likelihood that those experiencing poor sleep quality may be at risk of developing physical complications, thus, reducing HRQoL in this patient cohort. These findings elucidate the indirect relationship between sleep and on QoL, as inadequate and poor sleep quality can negatively influence biochemical factors that are critical to maintain glycaemic control. Our results are consistent with previous findings, which also demonstrated that insufficient and poor sleep were detrimental to various health aspects, including glycaemic control (Knutson, 2007; Lee et al., 2017). Further research, particularly longitudinal, is needed to determine the extent in which sleep may impact QoL through glycaemic control among T2D patients.

The burden of living with T2D may often be exacerbated by the presence of comorbid conditions. Research suggests a high prevalence of comorbidity, with estimates of 88.5% (Adriaanse et al., 2016), and 97.5% (Iglay et al., 2016) among patients across the literature. Our findings demonstrate a significant association between comorbidities and poor HRQoL, with patients presenting with multi and co-morbidity more likely than their counterparts to elicit poor QoL (Azharuddin et al., 2020; Luyster and Dunbar-Jacob, 2011). The complexity of this relationship can be understood when considering the impact of sleep, as poor sleep quality was associated with a higher number of comorbidities. Furthermore, a negative association was demonstrated between sleep disorders and physical QoL (Naranjo et al., 2020). This, once again, highlight the intricacies of the relationship between sleep and QoL – sleep quality may exacerbate comorbid conditions, thus, reducing QoL in this patient cohort.

### Strengths and limitations

Overall, this systematic review demonstrated an emergent body of good quality literature examining the relationship between sleep and QoL in type 2 diabetes patients. Included studies derived from several different countries and across continents, including North and South America, Europe and Asia, which increases the generalizability of findings across cultures. While there was considerable heterogeneity across outcome measures, all scales were validated and reliably assessed QoL among patients and in clinical practice (Jacobson et al., 1994; Jain et al., 2017; Ware and Sherbourne, 1992), which can elicit generalizability of findings. A further strength of the current review is the methodology, which involved the adoption of a dual screening and quality appraisal process. Both the primary and secondary reviewer screened titles and abstracts, full-texts and carried out quality appraisal separately to minimize bias that may result should only one individual carry out the systematic review.

While our review has helped to clarify the relationship between sleep and QoL in T2D patients, and quantify the prevalence of sleep problems in this cohort, there are several limitations within included studies that must be considered. A main feature across studies was the utilization of self-report measures in the assessment of sleep. An overarching issue in the context of self-reports is the credibility of such measurements. Even if participants are attempting to be as forthright and insightful, their self-reports are subject to various sources of inaccuracy, such as memory (Paulhus and Vazire, 2007). Despite this, sleep quality is arguably subjective in nature. To address this limitation, future studies should aim to employ both self-report and objective measures of sleep. A second limitation across included studies was the distinct lack of longitudinal research. No study included in our review adapted this design, which is necessary to determine the relationship between variables over time, and draw conclusions regarding causation.

There are also some limitations inherent in our methodology. Due to the heterogeneity across outcome measures, a narrative synthesis was employed. While this method elicits understanding and allows for interpretation and critique of the data (Greenhalgh et al., 2018), meta-analyses are often considered the gold standard for reducing bias while synthesizing quantitative data. Thus, the review method may limit generalizability of findings as study quality and effect size were not considered in the aggregation of findings to answer the review question. Furthermore, study requirements for inclusion within the review were published in a peer reviewed journal and published in English. These inclusion criteria can introduce both publication and language bias, which may in turn reduce the validity and generalizability of findings. Despite these limitations, our synthesis provides a novel insight into the direct and indirect relationship between sleep and QoL, which is critical to inform research and clinical practice.

### Implications for clinical practice

The current findings are clinically relevant as they elucidate the extent in which T2D patients suffer from sleep problems, and also demonstrate the detrimental effects insufficient and poor sleep quality have on HRQoL. Clarification on the nature of the relationship between sleep and QoL can assist with the implementation of effective interventions that reduce sleep problems and improve sleep quality, thus enhancing QoL among patients. Given the high rates of sleep problems reported by T2D patients, and the detrimental effects they may have on QoL, there is an urgent need for the implementation of targeted solutions to alleviate this problem. The identification of a direct relationship between sleep and QoL creates an opportunity to develop targeted sleep interventions that may enhance QoL in this patient cohort. The referral of patients to sleep specialists for the treatment of disordered sleep, and the incorporation of sleep hygiene as part of diabetes-management could prove efficacious in alleviating symptoms and increasing patient QoL.

### Implications for future research

Future research could explore the impact of our suggestion that the impact of sleep hygiene be incorporated in clinical practice. Additionally, there is a striking need to align QoL outcome measures in order to allow for greater comparison and perhaps meta-analysis. Finally, some longitudinal research would be particularly helpful in further elucidating the nature of the relationship between sleep and quality of life in T2D patients.

## Conclusion

Our narrative synthesis has clarified the direct and indirect relationship between sleep and QoL among T2D patients. Clarification on this relationship gives rise to a number of avenues for potential treatment of this problem among patients, such as the development of targeted interventions to alleviate sleep symptoms and improve sleep quality. Our synthesis also emphasizes the importance of screening for sleep problems in T2D patients, as doing so could enable referral to a sleep specialist if appropriate. Furthermore, the incorporation of sleep hygiene into diabetes self-management could increase patient QoL directly through improved sleep quality, and indirectly through the reduction of sleep problems that exacerbate symptoms of psychological disorders and comorbid conditions among patients. While we report a sufficient quantity of good quality research in this area, a distinct lack of longitudinal research inhibits conclusions regarding causation in this relationship. Future research should focus on bridging this gap through longitudinal research and intervention studies targeting sleep problems in T2D patients with the goal of improving QoL.

## Research Data

sj-csv-1-hpq-10.1177_13591053221140805 – for Understanding the relationship between sleep and quality of life in type 2 diabetes: A systematic review of the literature Click here for additional data file. sj-csv-1-hpq-10.1177_13591053221140805 for Understanding the relationship between sleep and quality of life in type 2 diabetes: A systematic review of the literature by Bróna Laverty, Sreelakshmi Puthezhath Jayanandan and Sinéad Smyth in Journal of Health Psychology This article is distributed under the terms of the Creative Commons Attribution 4.0 License ( http://www.creativecommons.org/licenses/by/4.0/) which permits any use, reproduction and distribution of the work without further permission provided the original work is attributed as specified on the SAGE and Open Access pages ( https://us.sagepub.com/en-us/nam/open-access-at-sage).

sj-docx-2-hpq-10.1177_13591053221140805 – Supplemental material for Understanding the relationship between sleep and quality of life in type 2 diabetes: A systematic review of the literature Click here for additional data file. Supplemental material, sj-docx-2-hpq-10.1177_13591053221140805 for Understanding the relationship between sleep and quality of life in type 2 diabetes: A systematic review of the literature by Bróna Laverty, Sreelakshmi Puthezhath Jayanandan and Sinéad Smyth in Journal of Health Psychology

sj-docx-3-hpq-10.1177_13591053221140805 – Supplemental material for Understanding the relationship between sleep and quality of life in type 2 diabetes: A systematic review of the literature Click here for additional data file. Supplemental material, sj-docx-3-hpq-10.1177_13591053221140805 for Understanding the relationship between sleep and quality of life in type 2 diabetes: A systematic review of the literature by Bróna Laverty, Sreelakshmi Puthezhath Jayanandan and Sinéad Smyth in Journal of Health Psychology

sj-docx-4-hpq-10.1177_13591053221140805 – Supplemental material for Understanding the relationship between sleep and quality of life in type 2 diabetes: A systematic review of the literature Click here for additional data file. Supplemental material, sj-docx-4-hpq-10.1177_13591053221140805 for Understanding the relationship between sleep and quality of life in type 2 diabetes: A systematic review of the literature by Bróna Laverty, Sreelakshmi Puthezhath Jayanandan and Sinéad Smyth in Journal of Health Psychology

sj-docx-5-hpq-10.1177_13591053221140805 – Supplemental material for Understanding the relationship between sleep and quality of life in type 2 diabetes: A systematic review of the literature Click here for additional data file. Supplemental material, sj-docx-5-hpq-10.1177_13591053221140805 for Understanding the relationship between sleep and quality of life in type 2 diabetes: A systematic review of the literature by Bróna Laverty, Sreelakshmi Puthezhath Jayanandan and Sinéad Smyth in Journal of Health Psychology

sj-docx-6-hpq-10.1177_13591053221140805 – Supplemental material for Understanding the relationship between sleep and quality of life in type 2 diabetes: A systematic review of the literature Click here for additional data file. Supplemental material, sj-docx-6-hpq-10.1177_13591053221140805 for Understanding the relationship between sleep and quality of life in type 2 diabetes: A systematic review of the literature by Bróna Laverty, Sreelakshmi Puthezhath Jayanandan and Sinéad Smyth in Journal of Health Psychology

## References

[bibr1-13591053221140805] Adriaanse MC Drewes HW van der Heide I , et al. (2016) The impact of comorbid chronic conditions on quality of life in type 2 diabetes patients. Quality of Life Research 25(1): 175–182. 26267523 10.1007/s11136-015-1061-0 PMC4706581

[bibr2-13591053221140805] American Diabetes Association (2017) 2. Classification and diagnosis of diabetes. Diabetes Care 40(Supplement_1): S11–S24. 10.2337/dc17-S005 27979889

[bibr3-13591053221140805] Azharuddin M Kapur P Adil M , et al. (2020) Health-related quality of life and sleep quality among North Indian type 2 diabetes mellitus patients: Evidence from a cross-sectional study. Sleep Medicine 73: 93–100. 32799030 10.1016/j.sleep.2020.04.022

[bibr4-13591053221140805] Bani-issa W Al-Shujairi AM Patrick L (2018) Association between quality of sleep and health-related quality of life in persons with diabetes mellitus type 2. Journal of Clinical Nursing 27(7–8): 1653–1661. 29266588 10.1111/jocn.14221

[bibr5-13591053221140805] Bironneau V Goupil F Ducluzeau PH , et al. (2017) Association between obstructive sleep apnea severity and endothelial dysfunction in patients with type 2 diabetes. Cardiovascular Diabetology 16(1): 39. 28327146 10.1186/s12933-017-0521-y PMC5361793

[bibr6-13591053221140805] Boles A Kandimalla R Reddy PH (2017) Dynamics of diabetes and obesity: Epidemiological perspective. Biochimica et Biophysica Acta (BBA) - Molecular Basis of Disease 1863(5): 1026–1036. 28130199 10.1016/j.bbadis.2017.01.016 PMC5429876

[bibr7-13591053221140805] Cappuccio FP D’Elia L Strazzullo P , et al. (2010) Quantity and quality of sleep and incidence of type 2 diabetes. Diabetes Care 33(2): 414–420. 19910503 10.2337/dc09-1124 PMC2809295

[bibr8-13591053221140805] Chasens ER Korytkowski M Sereika SM , et al. (2013) Effect of poor sleep quality and excessive daytime sleepiness on factors associated with diabetes self-management. The Diabetes Educator 39(1): 74–82. 23192600 10.1177/0145721712467683 PMC3677551

[bibr9-13591053221140805] Chasens ER Sereika SM Burke LE , et al. (2014) Sleep, health-related quality of life, and functional outcomes in adults with diabetes. Applied Nursing Research 27(4): 237–241. 24746284 10.1016/j.apnr.2014.02.006 PMC4147025

[bibr10-13591053221140805] Chatterjee S Khunti K Davies MJ (2017) Type 2 diabetes. The Lancet 389(10085): 2239–2251. 10.1016/S0140-6736(17)30058-2 28190580

[bibr11-13591053221140805] Chen A Zhang ZL Liao ZH , et al. (2006) Self-management and quality of life in patients with diabetes mellitus. Chinese Journal Behavioural Medical Science 15(5): 434–436.

[bibr12-13591053221140805] Cho Y (2020) Early development of bidirectional associations between sleep disturbance and diabetes. Diabetes & Metabolism Journal 44(5): 668–670. 33115210 10.4093/dmj.2020.0198 PMC7643604

[bibr13-13591053221140805] Colten HR Altevogt BM (2006) Sleep Disorders and Sleep Deprivation: An Unmet Public Health Problem. Washington, DC: Institute of Medicine. 20669438

[bibr14-13591053221140805] Corporation for Digital Scholarship (2021) Zotero (Version 5.0) [Mac]. Available at: https://www.zotero.org/download/ (accessed 14 December 2022).

[bibr15-13591053221140805] Daniele TM de Bruin VM de Forte AC , et al. (2013) The relationship between physical activity, restless legs syndrome, and health-related quality of life in type 2 diabetes. Endocrine 44(1): 125–131. 23203003 10.1007/s12020-012-9841-6

[bibr16-13591053221140805] The DCCT Research Group (1988) Reliability and validity of a diabetes quality-of-life measure for the diabetes control and complications trial (DCCT). Diabetes Care 11(9): 725–732. 3066604 10.2337/diacare.11.9.725

[bibr17-13591053221140805] Dong D Lou P Wang J , et al. (2020) Interaction of sleep quality and anxiety on quality of life in individuals with type 2 diabetes mellitus. Health and Quality of Life Outcomes 18(1): 150. 32448338 10.1186/s12955-020-01406-z PMC7247196

[bibr18-13591053221140805] Dunne S Mooney O Coffey L , et al. (2017) Psychological variables associated with quality of life following primary treatment for head and neck cancer: A systematic review of the literature from 2004 to 2015. Psycho-Oncology 26(2): 149–160. 26918648 10.1002/pon.4109

[bibr19-13591053221140805] The EuroQol Group (1990) Euroqol: A new facility for the measurement of health-related quality of life. Health Policy 16(3): 199–208. 10109801 10.1016/0168-8510(90)90421-9

[bibr20-13591053221140805] Felce D Perry J (1995) Quality of life: Its definition and measurement. Research in Developmental Disabilities 16(1): 51–74. 7701092 10.1016/0891-4222(94)00028-8

[bibr21-13591053221140805] Gabric K Matetic A Vilovic M , et al. (2018) Health-related quality of life in type 2 diabetes mellitus patients with different risk for obstructive sleep apnea. Patient Preference and Adherence 12: 765–773. 29785091 10.2147/PPA.S165203 PMC5953311

[bibr22-13591053221140805] Gomersall T Madill A Summers LK (2011) A metasynthesis of the self-management of type 2 diabetes. Qualitative Health Research 21(6): 853–871. 21429946 10.1177/1049732311402096

[bibr23-13591053221140805] Greenhalgh T Thorne S Malterud K (2018) Time to challenge the spurious hierarchy of systematic over narrative reviews? European Journal of Clinical Investigation 48(6): e12931. 10.1111/eci.12931 PMC6001568 29578574

[bibr24-13591053221140805] Gupta S. Wang Z . (2016). Predictors of sleep disorders among patients with type 2 diabetes mellitus. Diabetes & Metabolic Syndrome: Clinical Research and Reviews, 10(4), 213–220. 10.1016/j.dsx.2016.06.009 27377685

[bibr25-13591053221140805] Hashimoto Y Sakai R Ikeda K , et al. (2020) Association between sleep disorder and quality of life in patients with type 2 diabetes: A cross-sectional study. BMC Endocrine Disorders 20(1): 98. 32605640 10.1186/s12902-020-00579-4 PMC7325681

[bibr26-13591053221140805] Iglay K Hannachi H Joseph Howie P , et al. (2016) Prevalence and co-prevalence of comorbidities among patients with type 2 diabetes mellitus. Current Medical Research and Opinion 32(7): 1243–1252. 26986190 10.1185/03007995.2016.1168291

[bibr27-13591053221140805] Jacobson AM Groot MD Samson JA (1994) The evaluation of two measures of quality of life in patients with type I and type II diabetes. Diabetes Care 17(4): 267–274. 8026281 10.2337/diacare.17.4.267

[bibr28-13591053221140805] Jain A Sharmab R Yadavc N , et al. (2017) Quality of life and its association with insomnia and clinical variables in type 2 diabetes. Journal of Egyptian Public Health Association 92(1): 52–59. 10.21608/epx.2018.7011 29924928

[bibr29-13591053221140805] Janzen Claude JA Hadjistavropoulos HD Friesen L (2013) Exploration of health anxiety among individuals with diabetes: Prevalence and implications. Journal of Health Psychology 19(2): 312–322. 23349403 10.1177/1359105312470157

[bibr30-13591053221140805] Johnson ST Thiel D Al Sayah F , et al. (2017) Objectively measured sleep and health-related quality of life in older adults with type 2 diabetes: A cross-sectional study from the Alberta’s caring for diabetes study. Sleep Health 3(2): 102–106. 28346155 10.1016/j.sleh.2016.12.002

[bibr31-13591053221140805] Khandelwal D. Dutta D. Chittawar S. Kalra S . (2017). Sleep disorders in type 2 diabetes. Indian Journal of Endocrinology and Metabolism, 21(5), 758. 28989888 10.4103/ijem.IJEM_156_17 PMC5628550

[bibr32-13591053221140805] Khuwaja A. K. Lalani S. Dhanani R. Azam I. S. Rafique G. White F . (2010). Anxiety and depression among outpatients with type 2 diabetes: A multi-centre study of prevalence and associated factors. Diabetology & Metabolic Syndrome, 2(1), 1–7. 21171976 10.1186/1758-5996-2-72 PMC3022608

[bibr33-13591053221140805] Kleefstra N Landman GWD Houweling ST , et al. (2008) Prediction of mortality in type 2 diabetes from health-related quality of life (zodiac-4). Diabetes Care 31(5): 932–933. 18319325 10.2337/dc07-2072

[bibr34-13591053221140805] Knol MJ Twisk JW Beekman AT , et al. (2006) Depression as a risk factor for the onset of type 2 diabetes mellitus. A meta-analysis. Diabetologia 49(5): 837–845. 16520921 10.1007/s00125-006-0159-x

[bibr35-13591053221140805] Knutson KL (2007) Impact of sleep and sleep loss on glucose homeostasis and appetite regulation. Sleep Medicine Clinics 2(2): 187–197. 18516218 10.1016/j.jsmc.2007.03.004 PMC2084401

[bibr36-13591053221140805] Kuo HJ Huang YC García AA (2022) An integrative review of fatigue in adults with type 2 diabetes mellitus: Implications for self-management and quality of life. Journal of Clinical Nursing 31(11–12): 1409–1427. 34585452 10.1111/jocn.16058

[bibr37-13591053221140805] Lee SW Ng KY Chin WK (2017) The impact of sleep amount and sleep quality on glycemic control in type 2 diabetes: A systematic review and meta-analysis. Sleep Medicine Reviews 31: 91–101. 26944909 10.1016/j.smrv.2016.02.001

[bibr38-13591053221140805] Li H Ji M Scott P , et al. (2019) The effect of symptom clusters on quality of life among patients with type 2 diabetes. The Diabetes Educator 45(3): 287–294. 30873908 10.1177/0145721719837902

[bibr39-13591053221140805] Lou P Qin Y Zhang P , et al. (2015) Association of Sleep Quality and quality of life in type 2 diabetes mellitus: A cross-sectional study in China. Diabetes Research and Clinical Practice 107(1): 69–76. 25458325 10.1016/j.diabres.2014.09.060

[bibr40-13591053221140805] Luyster FS Dunbar-Jacob J (2011) Sleep quality and quality of life in adults with type 2 diabetes. The Diabetes Educator 37(3): 347–355. 21467248 10.1177/0145721711400663 PMC3220408

[bibr41-13591053221140805] Manber R Edinger JD Gress JL , et al. (2008) Cognitive behavioral therapy for insomnia enhances depression outcome in patients with comorbid major depressive disorder and insomnia. Sleep 31(4): 489–495. 18457236 10.1093/sleep/31.4.489 PMC2279754

[bibr42-13591053221140805] Martins RC Andersen ML Tufik S (2008) The reciprocal interaction between sleep and type 2 diabetes mellitus: Facts and perspectives. Brazilian Journal of Medical and Biological Research 41(3): 180–187. 18060321 10.1590/s0100-879x2006005000194

[bibr43-13591053221140805] Merlino G Valente M Serafini A , et al. (2010) Effects of restless legs syndrome on quality of life and psychological status in patients with type 2 diabetes. The Diabetes Educator 36(1): 79–87. 20185611 10.1177/0145721709351252

[bibr44-13591053221140805] Modarresnia L Golgiri F Madani NH , et al. (2018) Restless legs syndrome in Iranian people with type 2 diabetes mellitus: The role in quality of life and quality of sleep. Journal of Clinical Sleep Medicine 14(02): 223–228. 29351820 10.5664/jcsm.6938 PMC5786841

[bibr45-13591053221140805] Moher D Liberati A Tetzlaff J , et al. (2009) Preferred reporting items for systematic reviews and meta-analyses: The Prisma statement. PLoS Medicine 6(7): e1000097. 10.1371/journal.pmed.1000097 PMC2707599 19621072

[bibr46-13591053221140805] Naranjo C Ortega-Jiménez P del Reguero L , et al. (2020) Relationship between diabetic neuropathic pain and comorbidity. their impact on pain intensity, diabetes complications and quality of life in patients with type-2 diabetes mellitus. Diabetes Research and Clinical Practice 165: 108236. 32470476 10.1016/j.diabres.2020.108236

[bibr47-13591053221140805] Narisawa H Komada Y Miwa T , et al. (2017) Prevalence, symptomatic features, and factors associated with sleep disturbance/insomnia in Japanese patients with type-2 diabetes. Neuropsychiatric Disease and Treatment 13: 1873–1880. 28765709 10.2147/NDT.S134814 PMC5525901

[bibr48-13591053221140805] Nathan DM Buse JB Davidson MB , et al. (2009) Medical Management of hyperglycemia in type 2 diabetes: A consensus algorithm for the initiation and adjustment of therapy: A consensus statement of the American Diabetes Association and the European Association for the study of diabetes. Diabetes Care 32(5): 193–203. 18945920 10.2337/dc08-9025 PMC2606813

[bibr49-13591053221140805] Nefs G. Donga E. van Someren E. Bot M. Speight J. Pouwer F . (2015). Subjective sleep impairment in adults with type 1 or type 2 diabetes: Results from Diabetes MILES—The Netherlands. Diabetes Research and Clinical Practice, 109(3), 466–475. 26264411 10.1016/j.diabres.2015.07.008

[bibr50-13591053221140805] Onu DU Ifeagwazi CM Prince OA (2022) Social support buffers the impacts of diabetes distress on health-related quality of life among type 2 diabetic patients. Journal of Health Psychology 27: 2305–2317. 33406922 10.1177/1359105320980821

[bibr51-13591053221140805] Pamidi S Tasali E (2012) Obstructive sleep apnea and type 2 diabetes: Is there a link? Frontiers in Neurology 3: 126. 23015803 10.3389/fneur.2012.00126 PMC3449487

[bibr52-13591053221140805] Paulhus DL Vazire S (2007) The self-report method. In Robins RW Fraley RC Krueger RF (eds) Handbook of Research Methods in Personality Psychology. New York, NY: Guilford Press, pp.224–239.

[bibr53-13591053221140805] Pouwer F (2009) Should we screen for emotional distress in type 2 diabetes mellitus? Nature Reviews Endocrinology 5(12): 665–671. 10.1038/nrendo.2009.214 19884900

[bibr54-13591053221140805] Rubin RR (2000) Diabetes and quality of life. Diabetes Spectrum 13(1): 21.

[bibr55-13591053221140805] Seligowski AV Pless Kaiser AP Niles BL , et al. (2013) Sleep quality as a potential mediator between psychological distress and diabetes quality of life in veterans with type 2 diabetes. Journal of Clinical Psychology 69(10): 1121–1131. 22638910 10.1002/jclp.21866

[bibr56-13591053221140805] Spiegel K Knutson K Leproult R , et al. (2005) Sleep loss: A novel risk factor for insulin resistance and type 2 diabetes. Journal of Applied Physiology 99(5): 2008–2019. 16227462 10.1152/japplphysiol.00660.2005

[bibr57-13591053221140805] Stolar M (2010) Glycemic control and complications in type 2 diabetes mellitus. The American Journal of Medicine 123(3): S3–S11. 10.1016/j.amjmed.2009.12.004 20206730

[bibr58-13591053221140805] Sun N Lou P Shang Y , et al. (2016) Prevalence and determinants of depressive and anxiety symptoms in adults with type 2 diabetes in China: A cross-sectional study. BMJ Open 6(8): e012540. 10.1136/bmjopen-2016-012540 PMC5013513 27531739

[bibr59-13591053221140805] Tovilla-Zárate C Juárez-Rojop I Peralta Jimenez Y , et al. (2012) Prevalence of anxiety and depression among outpatients with type 2 diabetes in the Mexican population. PLoS One 7(5): e36887. 10.1371/journal.pone.0036887 PMC3356343 22629339

[bibr60-13591053221140805] Trikkalinou A Papazafiropoulou AK Melidonis A (2017) Type 2 diabetes and quality of life. World Journal of Diabetes 8(4): 120–129. 28465788 10.4239/wjd.v8.i4.120 PMC5394731

[bibr61-13591053221140805] Veritas Health Innovation (2021) Covidence systematic review soft- ware [Mac]. Melbourne, Australia. Available at www.covidence.org (accessed 14 December 2022).

[bibr62-13591053221140805] Vieira V Verussa T Lagacci M , et al. (2008) Quality of sleep and quality of life in people with type 2 diabetes. Journal of Diabetes Nursing 12(7): 262–270.

[bibr63-13591053221140805] Ware JE Sherbourne CD (1992) The MOS 36-ltem short-form health survey (SF-36). Medical Care 30(6): 473–483. 1593914

[bibr64-13591053221140805] World Health Organization (1996) WHOQOL-BREF: Introduction, administration, scoring and generic version of the assessment: Field trial version, December 1996 (No. WHOQOL-BREF). Geneva, Switzerland: World Health Organization.

[bibr65-13591053221140805] Wu Y Ding Y Tanaka Y , et al. (2014) Risk factors contributing to type 2 diabetes and recent advances in the treatment and prevention. International Journal of Medical Sciences 11(11): 1185–1200. 25249787 10.7150/ijms.10001 PMC4166864

[bibr66-13591053221140805] Yaggi HK Araujo AB McKinlay JB (2006) Sleep duration as a risk factor for the development of type 2 diabetes. Diabetes Care 29(3): 657–661. 16505522 10.2337/diacare.29.03.06.dc05-0879

[bibr67-13591053221140805] Yücel ŞÇ Güler EK Ak İ (2015) Investigation of sleep quality, quality of life, anxiety and depression in patients with diabetes mellitus. International Journal of Diabetes in Developing Countries 35(1): 39–46.

[bibr68-13591053221140805] Zeng Y Wu J Yin J , et al. (2018) Association of the combination of sleep duration and sleep quality with quality of life in type 2 diabetes patients. Quality of Life Research 27(12): 3123–3130. 30030675 10.1007/s11136-018-1942-0

[bibr69-13591053221140805] Zhang P Lou P Chang G , et al. (2016) Combined effects of sleep quality and depression on quality of life in patients with type 2 diabetes. BMC Family Practice 17(1): 1–7. 27044393 10.1186/s12875-016-0435-x PMC4820902

[bibr70-13591053221140805] Zhao J Li X-L Han K , et al. (2016) Biological interaction between sleep quality and depression in type 2 diabetes. European Review for Medical and Pharmacological Sciences 20(14): 3087–3091. 27460739

[bibr71-13591053221140805] Zhu B Vincent C Kapella MC , et al. (2018) Sleep disturbance in people with diabetes: A concept analysis. Journal of Clinical Nursing 27(1–2): e50–e60. 10.1111/jocn.14010 PMC6873709 28793386

