# Supplementary material for: Understanding the relationship between sleep and quality of life in type 2 diabetes: A systematic review of the literature
Source: J Health Psychol. 2023 Jan 4;28(8):693–710. doi: 10.1177/13591053221140805 (PMC10291116; doi:10.1177/13591053221140805)
Supplement: sj-docx-2-hpq-10.1177_13591053221140805 – Supplemental material for Understanding the relationship between sleep and quality of life in type 2 diabetes: A systematic review of the literature [file sj-docx-2-hpq-10.1177_13591053221140805.docx]

The data related to this publication are as follows:

1. A separate PDF file containing the syntax for the searches on all data bases.
2. A separate CSV file containing the “raw data” or the full list of studies returned by the search and which were screened at the abstract stage.
3. The data extracted from each of the included studies which is within the manuscript (Table 2).
4. The results of the quality appraisal process which is included within the manuscript (Table 3).
