# Supplementary material for: Understanding the relationship between sleep and quality of life in type 2 diabetes: A systematic review of the literature
Source: J Health Psychol. 2023 Jan 4;28(8):693–710. doi: 10.1177/13591053221140805 (PMC10291116; doi:10.1177/13591053221140805)
Supplement: sj-docx-3-hpq-10.1177_13591053221140805 – Supplemental material for Understanding the relationship between sleep and quality of life in type 2 diabetes: A systematic review of the literature [file sj-docx-3-hpq-10.1177_13591053221140805.docx]

**Appendix B**

*PICOS Table of Study Inclusion and Exclusion Criteria*

| **PICO** | **Inclusion criteria** | **Exclusion criteria** |
| --- | --- | --- |
| Population/participants | - Participants over the age of 18, any gender - Participants must have a diagnosis of type 2 diabetes | - Studies with participants under the age of 18 - Patients with T1D - Patients with gestational diabetes - Patients with pancreatogenic diabetes |
| Intervention/issue | - Studies with sleep as the independent variable - Sleep may also be a dependent variable - Studies examining the effect of disordered sleep, sleep disturbances, sleep quality and sleep duration on quality of life | - Studies in which sleep is not the independent variable - Studies in which quality of life is not included as a dependent variable - Studies with a sleep intervention |
| Comparator | N/A | N/A |
| Outcome | - Primary outcome is quality of life among T2DM patients. - Validated measures of quality of life to be included are; quality of life, health-related quality of life and diabetes-related quality of life. - Administered or self-reported measures of quality of life. | - Measures which are not valid assessments of quality of life |
| Study design | - Studies can be cross-sectional, longitudinal or experimental - May employ the use of surveys - Quantitative or mixed methods - Empirical research studies - Papers must be published in peer reviewed journal - Published in the English language - Papers must quantitatively measure sleep and QoL - Full-text articles must be available through database or through contact with authors   Studies that recruit both T1D and T2D patients that differentiate findings between both cohorts | - Papers utilising only qualitative research methods - Papers with qualitative study designs such as interview and focus groups - Not published in a peer-reviewed journal - Grey literature - Published in any language other than English - Full-text article not available - Studies that recruit participants with both T1D and T2D but do   not differentiate findings between cohorts |
