# Supplementary material for: Understanding the relationship between sleep and quality of life in type 2 diabetes: A systematic review of the literature
Source: J Health Psychol. 2023 Jan 4;28(8):693–710. doi: 10.1177/13591053221140805 (PMC10291116; doi:10.1177/13591053221140805)
Supplement: sj-docx-4-hpq-10.1177_13591053221140805 – Supplemental material for Understanding the relationship between sleep and quality of life in type 2 diabetes: A systematic review of the literature [file sj-docx-4-hpq-10.1177_13591053221140805.docx]

**Appendix C**

*Outcome of Quality Appraisal*

| **Criteria** | Azharuddin et al (2020) | Bani-Issa et al (2017) | Bironneau et al (2017) | Chasens et al (2014) | Daniele et al (2013) | Dong et al (2020) | Gabric et al (2018) | Hashimoto et al (2020) | Jain et al (2017) | Johnson et al (2017) | Li et al (2019) | Lou et al (2015) |
| --- | --- | --- | --- | --- | --- | --- | --- | --- | --- | --- | --- | --- |
| Clearly stated aims | Y | Y | Y | Y | Y | Y | Y | Y | Y | Y | Y | Y |
| Participant eligibility and recruitment strategy clearly documented | Y | Y | Y | Y | Y | Y | Y | Y | Y | Y | Y | Y |
| Main features of population/design described | Y | Y | Y | Y | Y | Y | Y | Y | Y | Y | Y | Y |
| Non-responders (and non-participants) described | Y | Y | P | N | N | Y | N | N | Y | P | N | Y |
| Presence of a control group | N | N | N | N | Y | N | N | N | N | N | Y | N |
| Main limitations identified and acceptable | Y | P | Y | Y | P | Y | P | P | P | Y | Y | Y |
| Sample size justified | N | Y | N | Y | N | Y | N | P | P | P | N | Y |
| No evidence of selective reporting of results | Y | Y | Y | Y | Y | Y | Y | P | Y | Y | Y | Y |
| Statistical methods described | Y | Y | Y | Y | Y | Y | Y | Y | Y | Y | Y | Y |
| Statistical methods appropriate | Y | Y | Y | Y | Y | Y | Y | Y | Y | Y | Y | Y |
| Measures relevant, validated and described adequately | Y | Y | Y | Y | Y | Y | Y | Y | Y | Y | Y | Y |
| Results discussed adequately | Y | Y | Y | Y | Y | Y | Y | P | Y | Y | Y | Y |
| **Total** | **20** | **21** | **19** | **20** | **19** | **22** | **17** | **16** | **18** | **20** | **20** | **22** |

**Table 3. Continued.**

*Dunne and Colleagues (2017) Quality Appraisal Tool*

| **Criteria** | Luyster et al (2011) | Merlino et al (2010) | Modarresnia et al (2018) | Naranjo et al (2020) | Narisawa et al (2017) | Seligowski et al (2013) | Vieira et al (2008) | Yücel et al (2015) | | Zeng et al (2018) | | Zhang et al (2016) | Zhao et al (2016) |
| --- | --- | --- | --- | --- | --- | --- | --- | --- | --- | --- | --- | --- | --- |
| Clearly stated aims | Y | Y | Y | Y | P | Y | Y | Y | Y | | Y | | P |
| Participant eligibility and recruitment strategy clearly documented | Y | Y | Y | Y | Y | Y | Y | P | P | | Y | | Y |
| Main features of population/design described | Y | P | Y | Y | Y | Y | Y | P | N | | Y | | Y |
| Non-responders (and non-participants) described | P | N | N | N | P | Y | N | N | N | | Y | | N |
| Presence of a control group | N | Y | N | Y | Y | N | N | N | N | | N | | N |
| Main limitations identified and acceptable | Y | Y | P | Y | Y | Y | P | N | Y | | Y | | N |
| Sample size justified | Y | P | Y | N | P | Y | N | N | P | | P | | N |
| No evidence of selective reporting of results | Y | Y | Y | Y | Y | Y | Y | Y | Y | | Y | | P |
| Statistical methods described | Y | Y | Y | Y | Y | Y | P | N | Y | | Y | | Y |
| Statistical methods appropriate | Y | Y | Y | Y | Y | Y | Y | P | Y | | Y | | Y |
| Measures relevant, validated and described adequately | Y | Y | Y | Y | Y | Y | Y | Y | P | | Y | | Y |
| Results discussed adequately | Y | Y | Y | Y | Y | Y | Y | Y | Y | | Y | | Y |
| **Total** | **21** | **20** | **19** | **20** | **21** | **22** | **16** | **11** | **15** | | **21** | | **14** |
