# Supplementary material for: Understanding the relationship between sleep and quality of life in type 2 diabetes: A systematic review of the literature
Source: J Health Psychol. 2023 Jan 4;28(8):693–710. doi: 10.1177/13591053221140805 (PMC10291116; doi:10.1177/13591053221140805)
Supplement: sj-docx-5-hpq-10.1177_13591053221140805 – Supplemental material for Understanding the relationship between sleep and quality of life in type 2 diabetes: A systematic review of the literature [file sj-docx-5-hpq-10.1177_13591053221140805.docx]

**Appendix A**

**Web of Science/MEDLINE/CINAHL/PsychINFO Search Terms**

Sleep

AND

“Quality of life” OR QOL OR “health-related quality of life” OR wellbeing OR well-being Or “diabetes-related quality of life” OR “diabetes related quality of life” OR “health related quality of life” OR “HRQOL”

AND

Diabetes OR “diabetes mellitus” OR diabetics OR diabetic
