# Supplementary material for: Understanding the relationship between sleep and quality of life in type 2 diabetes: A systematic review of the literature
Source: J Health Psychol. 2023 Jan 4;28(8):693–710. doi: 10.1177/13591053221140805 (PMC10291116; doi:10.1177/13591053221140805)
Supplement: sj-docx-6-hpq-10.1177_13591053221140805 – Supplemental material for Understanding the relationship between sleep and quality of life in type 2 diabetes: A systematic review of the literature [file sj-docx-6-hpq-10.1177_13591053221140805.docx]

The data related to this publication are as follows:

1. A separate PDF file containing the syntax for the searches on all databases.
2. A separate CSV file containing the “raw data” or the full list of studies returned by the search and which were screened at the abstract stage.
3. A separate PDF file containing the inclusion criteria for studies included in the review.
4. A separate PDF file containing results of the quality appraisal process which is included within the manuscript.
5. A PRISMA diagram outlining the studies returned and excluded at each stage of the review, which is within the manuscript (Figure 1).
6. The data extracted from each of the included studies, which is within the manuscript (Table 1).

An exact replication of the systematic review will not be possible given that additional relevant manuscripts will be indexed regularly. However, an updated version can be run by using the syntax supplied in order to search on the named databases. Following the detailed procedure provided in the manuscript, the titles and abstracts can be screened by two independent reviews and then, full-texts can also be screened independently. We used Covidence to facilitate this. Using the headings provided in Table 2 of the manuscript, data can be extracted from each of the included studies.
